# International symposium on integrative bioinformatics 2024 – editorial

**DOI:** 10.1515/jib-2024-0051

**Published:** 2024-11-07

**Authors:** Can Türker, Christian Panse, Bjorn Sommer, Marcel Friedrichs, Ralf Hofestädt

**Affiliations:** Functional Genomics Center Zurich, University of Zurich/ETH Zurich, Winterthurerstrasse 190, 8057 Zurich, Switzerland; School of Design, Royal College of Art, Kensington Gore, SW7 2EU London, UK; Bioinformatics Department, Faculty of Technology, Bielefeld University, D-33501 Bielefeld, Germany

**Keywords:** integrative bioinformatics, modelling, simulation, visualization, genomics

## Abstract

Integrative Bioinformatics faces the challenge of integrating, aligning, modelling, and simulating data in a coherent fashion to gain deeper insights into complex biological systems. This special issue of the Journal of Integrative Bioinformatics consists of six articles accepted for the presentation at the “18th International Symposium on Integrative Bioinformatics” held in Zürich on September 12–13, 2024. In addition, the symposium featured five keynote talks which will be discussed here as well.

## 1 Introduction

**Figure 1:**
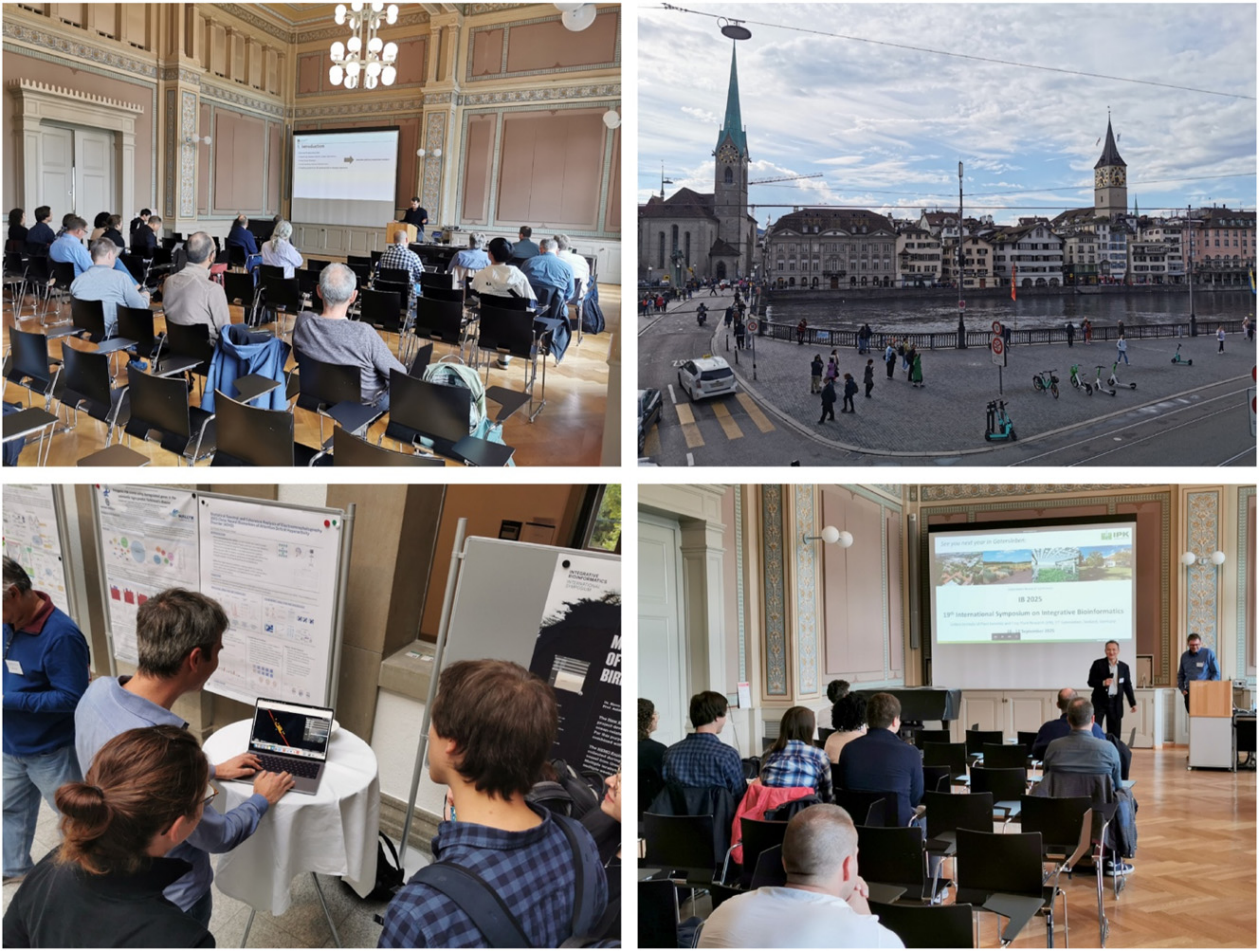
IB 2024 symposium: Top-left: the conference room at the Aula in the Rämistrasse 59 in Zurich; Top-right: attendees exploring Zurich around the Münsterbrücke with the two churches Fraumünster and St. Peter; Bottom-left: poster session; Bottom-right: closing remarks by the conference chairs Can Türker and Christian Panse.

Integrative Bioinformatics faces the challenge of integrating, aligning, modelling, and simulating data in a coherent fashion to gain deeper insights into complex biological systems. This special issue of the Journal of Integrative Bioinformatics consists of articles accepted for the presentation at the “18th International Symposium on Integrative Bioinformatics” held in Zürich on September 12–13, 2024. Thanks to all authors for contributing to this year’s event with their submitted articles. By addressing very different topics of the field, they laid the foundation for a highly interesting symposium (Figure 1). More details available here [1]: https://www.imbio.de/ib2024.

## 2 Proceedings

In “Leonhard Med, a trusted research environment for processing sensitive research data”, Michal Okoniewski et al. highlight the secure handling of sensitive research data through robust security measures and compliance with data protection standards. The paper also covers the platform’s evolution, design history, current status, and operations, emphasizing its role in supporting personalized medicine and genomic data analysis. It showcases pioneering work that has been successfully implemented and utilized by a growing community, addressing real-world integration challenges in handling human-sensitive data [2].

In “Exploring Animal Behaviour Multilayer Networks in Immersive Environments – a Conceptual Framework”, Stefan Feyer et al. introduce a concept for the visualization, exploration and analysis of animal behavior multilayer networks in immersive stereoscopic 3D environments, such as virtual reality (VR) or augmented reality (AR). They describe how the design space of multilayer networks could be arranged to meet the requirements of immersive environments [3].

The article “Layout of anatomical structures and blood vessels based on the foundational model of anatomy” by Niklas Gröne et al. showcases a novel approach to the visualization of the Foundational Model of Anatomy (FMA) ontology. It focuses on the integration and layout of vascular systems within a hierarchical anatomical framework with the goal to enhance the clarity and comprehension of complex anatomical and vascular data [4].

In “Constructing networks for comparison of collagen types” Valentin Wesp et al. analyze collagen families with different levels of conservation to increase the accuracy of collagen classification and prediction of their functions. It is highlighted that the structure of these collagens and their expression in different tissues could result in a better focus on sequence segments of interest and thus help to construct informative networks [5].

The article “Inferences on the evolution of the ascorbic acid synthesis pathway in insects using Phylogenetic Tree Collapser” by Daniel Glez-Pena et al. presents a flexible tool for automated tree collapsing using taxonomic information, which can easily be used by researchers without any computer science background. The utility of this tool is demonstrated by addressing the evolution of the ascorbic acid synthesis pathway in insects [6].

And finally, in the article “A roadmap for a middleware as a federation service for integrative data retrieval of agricultural data” by Jorge García Brizuela et al., the authors introduce a plan forward for the FAIRagro middleware infrastructure. Being part of the National Research Data Infrastructure (NFDI) in Germany, FAIRagro aims to connect existing data infrastructures which are associated with the agrosystems science community developing FAIR compliant infrastructure services [7].

## 3 Keynotes

In addition to the regular articles, the symposium featured five keynote speeches:

– Etzard Stolte provided an overview over the “AI & Knowledge Management” efforts at Roche Inc., highlighting the importance of data quality and integration for deriving knowledge.
– Natasha Glover, co-leader of the Swiss Institute of Bioinformatics (SIB) Comparative Genomics group, demonstrated the OMA browser and showed how this tool can be used for comparative genomics across the tree of life.
– Susanna Weber, the Data Steward Coordinator of the University of Zurich (UZH), presented FAIR Initiatives at UZH and outlined the different implementations of the data stewardship concept within Swiss universities.
– Menna El-Assady, from the ETH Zürich’s Interactive Visualization and Intelligence Augmentation Lab (IVIA), explored visualization tools for decision support in text mining on bridging the interface between humans and computers.
– Alexander Sczyrba, Forschungszentrum Jülich Centre for Biotechnology (CeBiTec), sketched projects supported by the German Network for Bioinformatics Infrastructure (de.NBI) and showcased methods for analysing large-scale metagenomics data.

The symposium concluded with a special acknowledgement to Ralf Hofestaedt, recognizing his contributions to the field of Bioinformatics as he approaches his forthcoming retirement.

We are pleased to announce that the 19th International Symposium on Integrative Bioinformatics is scheduled to be held at the Leibniz Institute of Plant Genetics and Crop Plant Research (IPK), OT Gatersleben, Seeland, Germany, from September 10–12, 2025. For more information, visit https://www.imbio.de/ib2025.

## References

[1] Panse C, Türker C, Friedrichs M (2024). IB2024 international symposium on integrative bioinformatics, 18th annual meeting.

[2] Okoniewski MJ, Wiegand A, Schmid DC, Bolliger C, Bovino C, Belluco M (2024). Leonhard med, a trusted research environment for processing sensitive research data. J Integr Bioinform.

[3] Feyer SP, Pinaud B, Klein K, Lein E, Schreiber F (2024). Exploring animal behaviour multilayer networks in immersive environments–a conceptual framework. J Integr Bioinform.

[4] Gröne N, Grüneisen B, Klein K, de Bono B, Czauderna T, Schreiber F (2024). Layout of anatomical structures and blood vessels based on the foundational model of anatomy. J Integr Bioinform.

[5] Wesp V, Scholz L, Ziermann-Canabarro JM, Schuster S, Stark H (2024). Constructing networks for comparison of collagen types. J Integr Bioinform.

[6] Glez-Peña D, López-Fernández H, Duque P, Vieira CP, Vieira J (2024). Inferences on the evolution of the ascorbic acid synthesis pathway in insects using phylogenetic tree collapser (PTC), a tool for the automated collapsing of phylogenetic trees using taxonomic information. J Integr Bioinform.

[7] Brizuela JG, Scharfenberg C, Scheuner C, Hoedt F, König P, Kranz A (2024). A roadmap for a middleware as a federation service for integrative data retrieval of agricultural data. J Integr Bioinform.

